# Environmental conditions and male quality traits simultaneously explain variation of multiple colour signals in male lizards

**DOI:** 10.1111/1365-2656.13773

**Published:** 2022-08-02

**Authors:** Arnaud Badiane, Andréaz Dupoué, Pauline Blaimont, Donald B. Miles, Anthony L. Gilbert, Mathieu Leroux‐Coyau, Anna Kawamoto, David Rozen‐Rechels, Sandrine Meylan, Jean Clobert, Jean‐François Le Galliard

**Affiliations:** ^1^ Sorbonne Université, CNRS, IRD, INRA Institut d'écologie et des sciences de l'environnement (IEES) Paris France; ^2^ Rider University Lawrenceville NJ USA; ^3^ Department of Biological Sciences Ohio University Athens OH USA; ^4^ Station d'Ecologie Théorique et Expérimentale (SETE), USR5321, CNRS Moulis France; ^5^ Centre de Recherche en Écologie Expérimentale et Prédictive (CEREEP‐Ecotron IleDeFrance) Département de biologie, Ecole Normale Supérieure CNRS, PSL Research University Saint‐Pierre‐lès‐Nemours France

**Keywords:** animal communication, colouration, parasitism, performance, reptile, testosterone, ultraviolet, *Zootoca vivipara*

## Abstract

1. Male lizards often display multiple pigment‐based and structural colour signals which may reflect various quality traits (e.g. performance, parasitism), with testosterone (T) often mediating these relationships. Furthermore, environmental conditions can explain colour signal variation by affecting processes such as signal efficacy, thermoregulation and camouflage. The relationships between colour signals, male quality traits and environmental factors have often been analysed in isolation, but simultaneous analyses are rare. Thus, the response of multiple colour signals to variation in all these factors in an integrative analysis remains to be investigated.
2. Here, we investigated how multiple colour signals relate to their information content, examined the role of T as a potential mediator of these relationships and how environmental factors explain colour signal variation.
3. We performed an integrative study to examine the covariation between three colour signals (melanin‐based black, carotenoid‐based yellow–orange and structural UV), physiological performance, parasitism, T levels and environmental factors (microclimate, forest cover) in male common lizards *Zootoca vivipara* from 13 populations.
4. We found that the three colour signals conveyed information on different aspects of male condition, supporting a multiple message hypothesis. T influenced only parasitism, suggesting that T does not directly mediate the relationships between colour signals and their information content. Moreover, colour signals became more saturated in forested habitats, suggesting an adaptation to degraded light conditions, and became generally brighter in mesic conditions, in contradiction with the thermal melanism hypothesis.
5. We show that distinct individual quality traits and environmental factors simultaneously explain variations of multiple colour signals with different production modes. Our study therefore highlights the complexity of colour signal evolution, involving various sets of selective pressures acting at the same time, but in different ways depending on colour production mechanism.

## Graphical abstract

Using the common lizard, Zootoca vivipara, this work shows that three different colour signals convey information on different aspects of male quality (performance, parasitism, body size), and at the same time covary with environmental factors such as forest cover, climate and ecogeography.

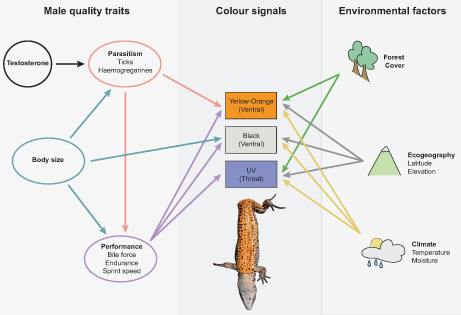

## Résumé

1. Les lézards mâles arborent souvent plusieurs signaux colorés de nature pigmentaire et structurale qui reflètent de multiples traits de qualité (e.g. performance, parasitisme), et la testostérone (T) joue souvent un rôle de médiateur dans ces relations. En outre, les conditions environnementales peuvent également expliquer les variations des signaux colorés en influençant des aspects tels que l'efficacité des signaux, la thermorégulation ou le camouflage. Les relations entre signaux colorés, traits de qualité individuelle et facteurs environnementaux ont souvent été analysées séparément, mais rarement de manière simultanée. Ainsi, la réponse de ces multiples signaux colorés aux variations de tous ces facteurs reste à explorer dans le contexte d'une étude intégrative.
2. Ici, nous explorons la relation entre ces multiples signaux colorés et leur contenu informatif, nous examinons le rôle de T comme médiateur potentiel de ces relations et nous recherchons si les conditions environnementales expliquent la variation de ces signaux colorés.
3. Nous avons mené une étude intégrative afin d'examiner la covariation entre trois types de signaux colorés (noir produit par la mélanine, jaune‐orange produit par les caroténoïdes et UV produit par des éléments structuraux), la performance physiologique, le parasitisme, les niveaux de T et les conditions environnementales (e.g. microclimat, couverture forestière) chez des mâles du lézard vivipare (*Zootoca vivipara*) provenant de 13 populations.
4. Nos résultats indiquent que les trois signaux colorés transmettent des informations sur différents aspects de la condition des mâles, en accord avec l'hypothèse de « messages multiples ». T influence uniquement le parasitisme, suggérant que T n'agit pas en tant que médiateur des relations entre ces signaux colorés et leur contenu informatif. De plus, les signaux colorés sont plus saturés dans les habitats les plus forestiers, ce qui suggère une adaptation à des conditions lumineuses dégradées. Enfin, les signaux colorés sont plus intenses lorsque les conditions sont mésiques, en contradiction avec l'hypothèse du mélanisme thermal.
5. Nous démontrons que différents traits de qualité individuelle et facteurs environnementaux expliquent de manière simultanée les variations de multiples signaux colorés impliquant différents modes de production. Notre étude souligne ainsi la complexité de l'évolution des signaux colorés, qui implique plusieurs types de pressions de sélection agissant en même temps mais dans des directions différentes selon le mode de production.

## 1 INTRODUCTION

The display of conspicuous colours is ubiquitous across species and their role in signalling, thermoregulation and camouflage has always intrigued ecologists (Cuthill et al., 2017). When they act as communication signals, colours evolve primarily in response to selection acting on the relationship between the signal's properties and its information content, and on its transmission efficacy towards the receiver (Bradbury & Vehrencamp, 2011), although thermoregulation and camouflage can also constrain colour signal expression (e.g. de Souza et al., 2016; Lindstedt et al., 2009). In the context of sexual selection, animals often display a mosaic of different colours that convey information about individual quality (Bradbury & Vehrencamp, 2011). Colour signals are produced from the deposition of pigments (e.g. melanin, carotenoids, pterins) in skin cells, from structural light‐scattering and light‐reflecting nanoelements, or both (Shawkey & D'Alba, 2017). These modes of production may entail differences in cost–benefit balances which may ultimately affect their design and function. For example, while carotenoid‐based colours are physiologically costly to produce (reviewed in Svensson & Wong, 2011), melanin‐based colour signals are thought to be associated mainly with social costs (reviewed in Roulin, 2016). However, the costs associated with pterin‐based and structural colour signals, if any, remain largely unknown (Andrade & Carneiro, 2021; Kemp et al., 2012), although social costs are potential candidates (e.g. Kawamoto et al., 2021). If we are to comprehend how colour signals become evolutionarily stable, it is critical to understand how the relationships among a colour signal, its information content and environmental factors vary based on the different production modes of multiple colour signals.

Many animals display multiple colour signals that convey information on various aspects of male quality during male–male competition and female mate choice (Endler & Mappes, 2017; Johnstone, 1996). Several hypotheses have been proposed to explain the evolution of multiple colour signals (Bro‐Jørgensen, 2010; Hebets & Papaj, 2005). The ‘multiple message’ hypothesis states that each signal component conveys a distinct information on male quality. In contrast, the ‘back‐up’ hypothesis suggests that multiple signals provide redundant information, thus compensating for signal unreliability (i.e. coding errors). Other hypotheses explain multiple signals via several receiver‐driven mechanisms, for example, having to do with enhancing conspicuousness (i.e. alerting signals, amplifiers), receiver psychology with multiple signals improving each other's discriminability or sensory overload (reviewed in Bro‐Jørgensen, 2010). Recent lines of arguments proposed that multiple signals coexist because they are shaped by dynamics selection regimes, for example, related to changing environments, sexually antagonistic coevolution or negative frequency‐dependent selection (Bro‐Jørgensen, 2010). In this context, some signals would be reliable in some circumstances, but not in others, and multiple colour signals may have evolved as an adaptive solution to these fluctuating regimes. Moreover, as colour patches often involve three‐dimensional integumentary structures containing multiple pigments and structural elements, Grether et al. (2004) underlined their potential to act as multicomponent signals, in which case they should be included within the ‘multiple signals’ framework described above.

Lizards are an ideal model to study the evolution of multiple colour signals, as melanin‐based, carotenoid‐based and structural colour signals often co‐occur on their body and have been shown to be honest signals of male quality, through correlations with fitness and assays of resource‐holding potential, such as bite force, locomotor capacity, body condition or parasite load (e.g. Martín & López, 2009; Pérez i de Lanuza et al., 2014; Plasman et al., 2015). Androgens such as testosterone (T) can regulate the expression of sexual signals and mediate their relationships with their information content through different pathways depending on colour production mechanisms. For instance, T can promote melanin‐based colour signals by stimulating melanophores or via pleiotropic effects (Roulin, 2016). T can also enhance structural colour signals (e.g. UV and blue) by increasing dermal melanisation under the iridophore layer (e.g. Quinn & Hews, 2003). Moreover, T can promote carotenoid‐based signals through its action on erythrophores (e.g. Kobayashi et al., 2009), by controlling carotenoids' acquisition and allocation (e.g. Peters, 2007), or regulating carotenoid‐related genes (e.g. Khalil et al., 2020). In parallel, T often influences male growth, leading to sexual size dimorphism (e.g. Cox et al., 2009) and can enhance some physiological traits such as endurance (e.g. Sinervo et al., 2000) and bite force (e.g. Husak et al., 2007). In addition, the immunocompetence handicap hypothesis (ICHH) posits that T plays a central role in colour signal evolution by maintaining signal honesty (Folstad & Karter, 1992). According to the ICHH, T secretions regulate colour signal expression, yet depress the immune system, thus making individuals more vulnerable to parasites. Elevated plasma T levels may also change male behaviour so as to increase exposure risks to parasites (e.g. Olsson et al., 2000). In fact, the relationship between T, immunity and parasite load can be more complex. Although T depresses immune functions (reviewed in Foo et al., 2017), its effect on parasite load seems to be conditional on parasite type (e.g. Fuxjager et al., 2011). Given all this, T is a good candidate to mediate the relationships between colour signals, their information content (e.g. condition, performance, parasite load) and their associated costs (e.g. parasitism). In contrast, the Hamilton–Zuk hypothesis (Hamilton & Zuk, 1982) predicts that bright colours correlate with low parasite load, thus signalling an individual's good genes, as a consequence of a genetic association between colour and immunity. To test these hypotheses, we require studies that include multiple colour signals (e.g. melanin‐based, carotenoid‐based, structural), various male quality traits, several parasite types and T levels.

Environmental conditions may also explain variation in colour signals among individuals and across species. First, environmental conditions can alter visual communication through variation in light intensity and spectral composition, and in background colour (e.g. Fleishman et al., 2020). For example, light conditions and background colour, which vary along forest cover gradients (Endler, 1993), shape colour signals in *Anolis* lizards such that signal detectability increases in its local environment (Fleishman et al., 2020; Leal & Fleishman, 2004). Second, environmental conditions can influence natural selection on animal colouration, for example, in relation to camouflage and thermoregulation (e.g. Stuart‐Fox & Moussalli, 2009). In ectothermic organisms, the thermal melanism hypothesis predicts that darker individuals heat their body up faster than lighter ones and should be favoured by natural selection in colder conditions (Clusella Trullas et al., 2007). Ecogeographical gradients in elevation and latitude, with their concomitant changes in climate known to constrain thermoregulation processes in ectotherms (e.g. Rozen‐Rechels et al., 2021), have produced colour signal variation consistent with the thermal melanism hypothesis (Reguera et al., 2014; Svensson & Waller, 2013). Similarly, Gloger's rule, as revised by Delhey (2019), predicts an association between humid environments and dark colouration (i.e. melanin deposition) in endotherms and possibly in ectotherms. Darker colouration is assumed to increase camouflage and protection against parasites. Whether these environmental factors simultaneously act on multiple colour signals with different production mechanism or independently influence specific colour signals remains an elusive question.

**FIGURE 1:**
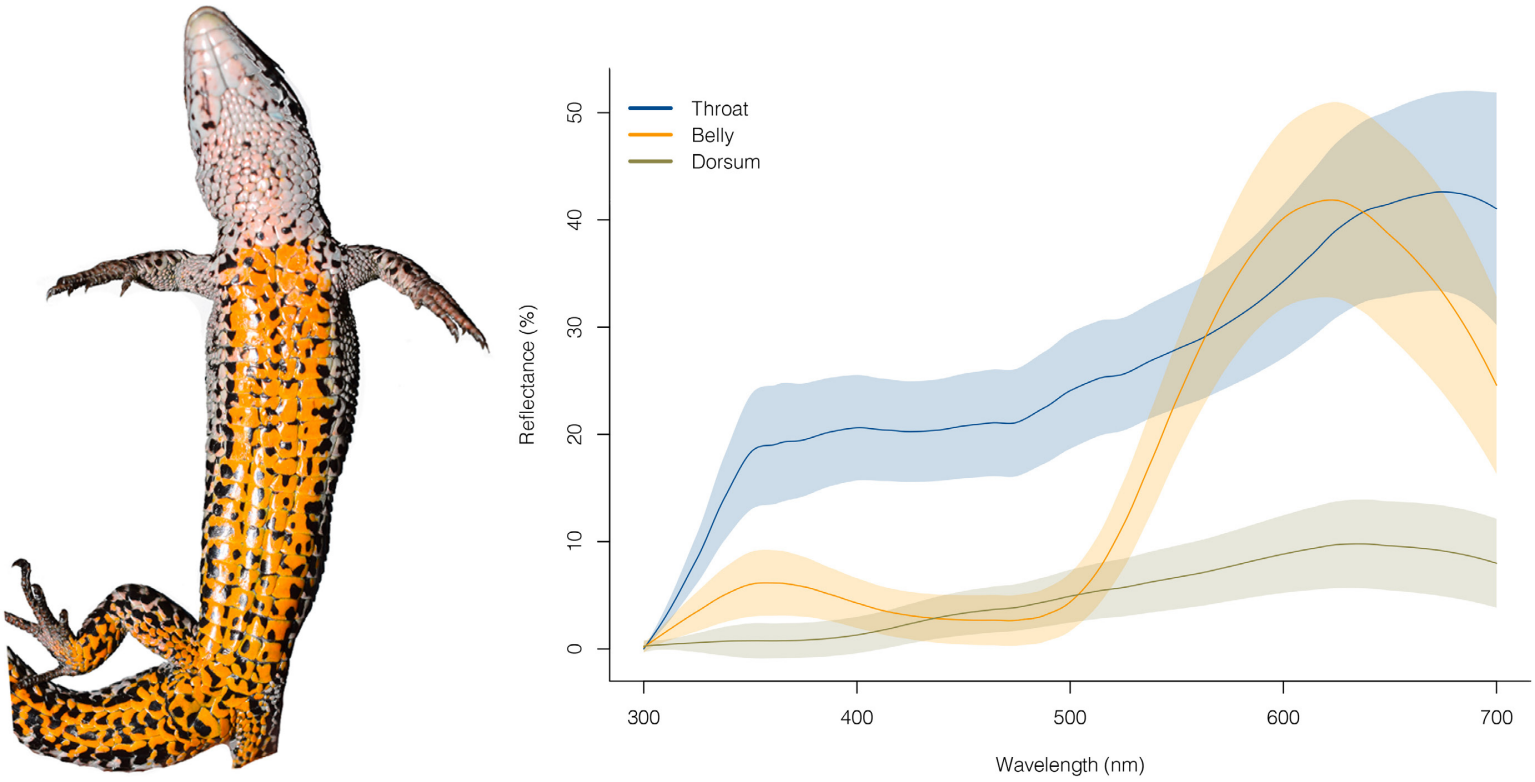
Ventral photograph of an adult male common lizard (left). Reflectance spectra from the throat, the venter and the dorsum of adult male common lizards between 300 and 700 nm, the typical visual range of common lizards (right). Lines represent the mean spectra from 211 individuals included in this study, and shaded areas represent the standard errors.

In this study, we used the common lizard *Zootoca vivipara* to shed light on the different factors explaining the variation of multiple colour signals. Male common lizards have a conspicuous, carotenoid‐based ventral colouration that ranges from yellow to orange, with a varying amount of melanic black spots dispersed along the venter, and a conspicuous UV‐white throat (Bonnaffé et al., 2018; Figure 1). The UV colouration on the throat plays a role during male–male competition (Martin et al., 2016) and female mate choice (Badiane et al., 2020), and is associated with social costs during male–male interactions (Kawamoto et al., 2021). Similarly, the amount of black ventral colouration correlates positively with male bite force, but decreases with sprint speed (San‐Jose et al., 2017). Finally, the carotenoid‐based yellow–orange ventral colouration which covaries with the physiological stress response is maintained by frequency‐dependent selection, and seems to play a role during female mate choice (Fitze et al., 2009; San‐Jose & Fitze, 2013).

We explored the degree to which these three components of ventral colouration with three distinct production mechanisms covary with individual quality traits, parasite load and T levels among individuals. We also examined how these colour components varied along environmental gradients, including habitat structure (forest cover), climate variables (temperature and moisture) and geographical variables (elevation and latitude) among several wild populations. Our goals are to: (a) explore how multiple colour signals relate to their potential information content and associated costs, (b) examine the role of T as a mediator of these relationships and (c) explore how environmental factors explain colour signal variation through their potential effect on signal efficacy and thermoregulation.

## 2 MATERIALS AND METHODS

### 2.1 Study species and populations

The European common lizard (*Zootoca vivipara*, family Lacertidae) is a small‐sized species inhabiting cool and mesic habitats across Eurasia. This species is polygynandrous with both males and females mating with multiple partners (Fitze et al., 2005). We sampled adult male lizards in 13 populations that differ in several ecogeographical components across the Massif Central mountain range in France (Supporting Information S1). We first recorded variables affecting habitat light conditions. We characterised the proportion of forest cover (mean = 33%, range = 0%–90%) for each population using Google Earth Pro landscape orthophotographs (2020 Google Image Landsat, Copernicus). Using the polygon tool, we surrounded each tree and group of trees from a given population and calculated the forested area in relation to the total population area. Forest cover is a direct index of habitat closeness, which can affect colour signal efficacy in lizards by modifying the ambient light environment (Endler, 1993; Leal & Fleishman, 2004). We also measured ambient light by taking irradiance spectra (i.e. 300–700 nm) from 20 random points at 12:00 pm under clear sky within each population using a JAZ spectrophotometer mounted with a cosine‐corrected sensor (Ocean Optics Inc.). In our study, the degree of forest cover has a weak negative correlation with mean total irradiance (i.e. 300–700 nm; *r* = 0.52, *p* < 0.001), but a large, negative correlation with mean irradiance UV chroma (i.e. the proportion of UV in the irradiance spectrum *I*_300–400_/*I*_300–700_; *r* = 0.95, *p* < 0.001) since foliage absorbs UV light (Endler, 1993). We included two ecogeographical variables, elevation and latitude, that could affect overall climate conditions and thus impose thermoregulation constraints on lizards (Rozen‐Rechels et al., 2021). We also recorded the mean annual temperatures and calculated a pluviometric quotient (i.e. *Q* Emberger, a measure of moisture conditions) using the same methods as Rozen‐Rechels et al. (2021) since both temperature and humidity are involved in thermoregulation (Rozen‐Rechels et al., 2021). High values of the *Q* index indicate more mesic conditions.

### 2.2 Morphology and colouration

In June 2018, we caught 211 adult males by hand from the 13 populations described above. We brought the lizards to a field laboratory and placed them in individual terraria for a maximum of 10 days. Individuals were given water ad libitum and live meal worms *Tenebrio molitor* every 2 days. On the day of capture, we weighed each lizard with a digital balance (to the nearest 0.01 g) and measured their snout–vent length (SVL in mm) with a ruler. We then obtained reflectance spectra from the belly and throat using a JAZ spectrophotometer (Ocean Optics Inc.) following the recommendations of Badiane et al. (2017) (Supporting Information S2). To quantify the ventral black colouration, we took calibrated (colour standards, millimetre scale) photographs in raw format of each lizard's venter using a Nikon D5300 DLSR with a Nikkor 40 mm Micro lens. We then used ImageJ (Schneider et al., 2012) to calculate the surface area covered by black colouration on the venter (i.e. absolute black), and the percentage of black colouration relative to the total ventral area (i.e. relative black).

### 2.3 Biomechanical performances

The next day, we measured bite force, a measure of fighting ability and whole‐organism performance in lizards (Lappin & Husak, 2005), using a purpose‐built bite force meter constructed from a modified Sauter 25N digital force gauge. We retained the maximum score out of three repeated measurements. After 2 days of rest, we estimated their locomotor performance at two ecologically relevant tasks: maximum sprint speed and endurance capacity. To measure endurance, we induced lizards to run on a motorised treadmill, by gently tapping their hindlimbs, at the constant speed of 0.5 km/hr until exhaustion. Exhaustion was assessed when lizards did not respond to three hindlimbs taps and lost their righting response (ability to flip over when placed on their back). To measure sprint speed, we induced lizards to sprint down a 1.5‐m racetrack lined with 25‐cm splits, and recorded the trials with a high‐speed camera (240 fps) placed above the racetrack. We analysed each trial frame by frame and retained the maximal sprint speed of the fastest 25‐cm section using the programme tracker. For logistical reasons, we were able to measure sprint speed for only 108 of the 211 individuals. Before each performance assay, we made sure the lizards had a body temperature close to 34°C during the tests (Rozen‐Rechels et al., 2020). Rozen‐Rechels et al. (2021) found minor differences in thermal preferences between our study populations (only 2% of the variance while inter‐individual differences explained 24% of the variance) and we therefore used the same body temperature for performance tests across all populations.

### 2.4 Plasma testosterone levels and parasitism

We collected blood samples (40–60 μl) from the infra‐orbital sinus using 20‐μl microhaematocrit tubes. We isolated the plasma by centrifugation and assayed plasma T levels using colorimetric competitive enzyme immunoassays (Testosterone ELISA kit, Cayman Chemical Cat, ref. 582701). On the day of capture, we also counted the number of ticks present on the body of each individual, an ectoparasite commonly found in common lizards (Wu et al., 2019). Ectoparasite infestation affects the life‐history traits, locomotor performance, dispersal behaviour and colouration of common lizards (Cote et al., 2010; Sorci & Clobert, 1995). We also quantified the prevalence of blood parasites from the haemogregarine group, which depends on environmental condijet al., 2013; Oppliger et al., 1996). To do so, we spread a drop of blood on a microscope slide and let it dry. We then fixated the slides into methanol for 60 s, let it dry and immersed them in modified Giemsa stain (GS500, Sigma‐Aldrich) for 45 min. Next, we used a microscope (×400) to count the number of blood cells infested by an haemogregarine parasite on the basis of 10,000 blood cells counted per individual.

### 2.5 Statistical analysis

We processed spectral data in R v.3.6.2 (R Development Core Team, 2017) using the package pavo 2.0 (Maia et al., 2019). We extracted the chromatic variables UV chroma (*R*_300–400_/*R*_300–700_) and luminance (*R*_300–700_) from the UV throat, and yellow–orange chroma (*R*_575–700_/*R*_300–700_) and luminance (*R*_300–700_) from the yellow–orange venter. To explore the relationships among colour traits, morphology, physiological performance, parasitism and plasmatic T levels, we used piecewise structural equation modelling (SEM) using the piecewiseSEM v2.1 package in r (Lefcheck, 2016) in combination with generalised linear mixed models using the r packages nlme (Pinheiro et al., 2019) and lme4 (Bates et al., 2015). SEM is a suitable tool to evaluate direct and indirect effects in descriptive analyses of complex systems (Grace et al., 2010). In addition, piecewise SEM tests for missing paths between variables using Shipley's test of d‐separation, which allows us to adjust the initial model to improve its fit and biological significance. Assessment of the goodness of fit of a model is first indicated by a non‐significant *p*‐value based on a Chi‐square test. Then, goodness of fit can be improved using a combination of indices, including Akaike's information criterion corrected for small sample size (AICc) obtained from Fisher's C statistic, and the Bayes–Schwarz information criterion (BIC).

We first built an initial model (detailed in Supporting Information S3) with physiological performance (i.e. endurance, bite force) and parasitism (i.e. number of ticks, number of haemogregarines) having direct effects on colour variation (i.e. throat UV chroma and luminance, ventral yellow–orange chroma and luminance, black area) as these variables are often involved in condition‐dependent signalling. We added a direct effect of body size on absolute black area based on the tests of d‐separation. We further set body size and T levels as direct drivers of the two physiological performance traits based on previous studies (Huyghe et al., 2009; John‐Alder et al., 2009) and further added a direct effect of haemogregarines on bite force based on d‐separation tests. We also tested a direct effect of T levels on body size (John‐Alder et al., 2009) and parasitism (Fuxjager et al., 2011). In addition, we specified correlated errors between our five colour variables, between the number of ticks and haemogregarines and between bite force and endurance. This allows the residual errors of two variables to be correlated for a reason not explained by our model when a direct causal effect is not biologically relevant. Both parasitism variables may reflect individual immunity and correlate with each other. Similarly, both performance variables may covary with other aspects of physiology and with each other.

We then discarded the non‐significant predictors until we obtained the lowest values of BIC. This piecewise SEM was performed on a sample size of 134 lizards, because we could not perform the most invasive protocols (performance scoring and blood sampling) in two populations subject to a long‐term mark–recapture survey and we had to exclude them from this part of the analysis (PIM and ROB in Supporting Information S1). In addition, we ran the same piecewise SEM using the relative black area instead of the absolute black area to test whether results differed. Furthermore, sprint speed was measured only on a subset of individuals and was thus analysed separately using GLMMs with the five colour variables as response (in five separate models); sprint speed and body size as predictor; and population as random intercept.

To explore the effects of ecogeographical variables on lizard colouration across all populations, we analysed variation in UV chroma, yellow–orange chroma, throat luminance, ventral luminance and absolute and relative area of black. We used GLMMs using the *lme* function in the r package nlme v3.1 (Pinheiro et al., 2019) with population identity as a random effect. We fitted a null model with only the intercept, and a model for every possible combination of the following predictors (only additive effects): elevation, latitude, forest cover, pluviometric quotient *Q* and T_mean_. We then proceeded with a model selection based on AICc, selecting only the best models (AICc < 2), and performed a conditional model averaging procedure using the r package MuMIn (Bartoń, 2019).

All continuous variables were mean‐centred–scaled by the standard deviation prior to analysing to improve the interpretation of the results (Schielzeth, 2010). We calculated a false discovery rate (FDR) of 15%, 20% and 25% following the Benjamini–Hochberg procedure to correct for multiple testing for both the SEM and model averaging analyses (Benjamini & Hochberg, 1995). All statistics of the SEM are summarised in Supporting Information S4 with FDR information available in Supporting Information S5. Analyses of ecophysiological variables are provided in Supporting Information S6 with FDR information available in Supporting Information S7.

### 2.6 Ethics statement

To capture common lizards *Zootoca vivipara* and collect all the data described above, we obtained authorisations from the French Ministry of Ecological Transition (ref TREL1734890A/6), the Cévennes National Parc (ref 2018‐0133) and the Ethics Committee for Animal Experimentation (ref 14574 2018040913579349 v4).

## 3 RESULTS

### 3.1 Colour signals and information content

**FIGURE 2:**
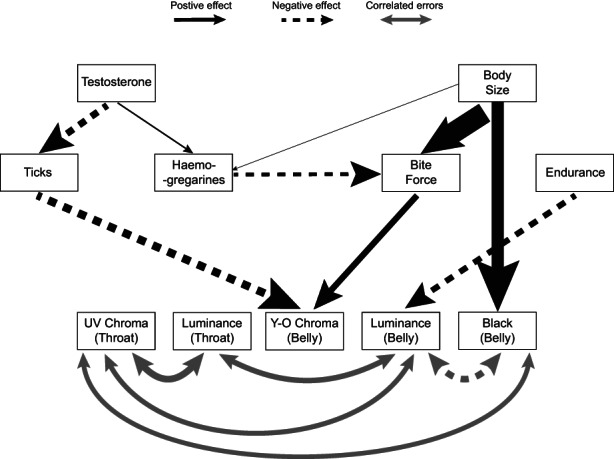
Best selected path diagram representing the relationships between colour signals (bottom line, 5 colour components), body size (measured by snout‐vent length), physiological performance (bite force and endurance), parasitism (number of ticks and blood haemogregarines) and plasma testosterone levels. Each single‐headed arrow represents a statistically significant direct causal path and arrow thickness is proportional to their effect size. Significant correlations are indicated by double header arrows.

The best model resulting from the piecewise SEM is presented in Figure 2. Our final model has a Fisher's *C* statistic of 32.135 with a *p*‐value of 0.46 and 32 degrees of freedom, implying that C conforms to what is expected given the number of degrees of freedom and that the model provided a good fit to our data. According to this best model, the number of haemogregarines increased with increasing T levels and body size (*R* ^2^_m_ = 0.04, *R* ^2^_c_ = 0.86). In contrast, the number of ticks decreased with increasing T levels (*R* ^2^_m_ = 0.06, *R* ^2^_c_ = 0.74). In addition, bite force covaried with body size, but was negatively affected by the number of haemogregarine parasites (*R* ^2^_m_ = 0.59, *R* ^2^_c_ = 0.62). We found no direct relationship between endurance and any other variables. Similarly, throat UV chroma is not influenced by bite force, endurance, parasitism or body size but correlates positively with both throat and ventral luminance (correlated residual errors).

**FIGURE 3:**
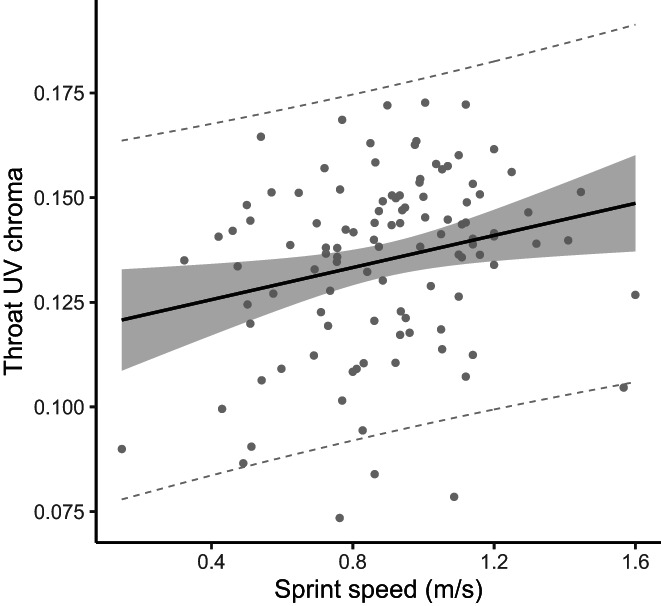
Best regression line between individual values of throat UV chroma (proportion of UV reflectance on the throat colour patch) and maximal sprint speed in male common lizards. Shaded area represents the 95% confidence interval and dashed lines represents the 95% prediction interval.

Furthermore, yellow–orange chroma increases with bite force and decreases with tick prevalence, but endurance does not have any significant influence (*R* ^2^_m_ = 0.16, *R* ^2^_c_ = 0.17). Ventral luminance is negatively affected by endurance, but not by tick prevalence (*R* ^2^_m_ = 0.07, *R* ^2^_c_ = 0.28), and correlates positively with throat luminance and UV chroma, but negatively with the amount of black colouration. Moreover, the amount of black colouration depends directly on body size (*R* ^2^_m_ = 0.21, *R* ^2^_c_ = 0.29) and correlates positively with throat UV chroma but negatively with ventral luminance. When we used the proportion of black colouration (%) instead of the absolute amount of black, the best model was qualitatively similar except that the proportion of black (%) is positively influenced by bite force instead of body size (*β* = 3.31 ± 1.66, standardised *β* = 0.17, *p* = 0.049, *R* ^2^_m_ = 0.03, *R* ^2^_c_ = 0.18; Path diagram showed in Supporting Information S8). In the sample of lizards measured for sprint speed, a separate LMM revealed that only throat UV chroma has a weak, positive correlation with sprint speed (*β* = 0.01 ± 0.01, *p* = 0.049, *R* ^2^_m_ = 0.04, *R* ^2^_c_ = 0.26—Figure 3).

### 3.2 Colour signals and ecogeographical variables

**FIGURE 4:**
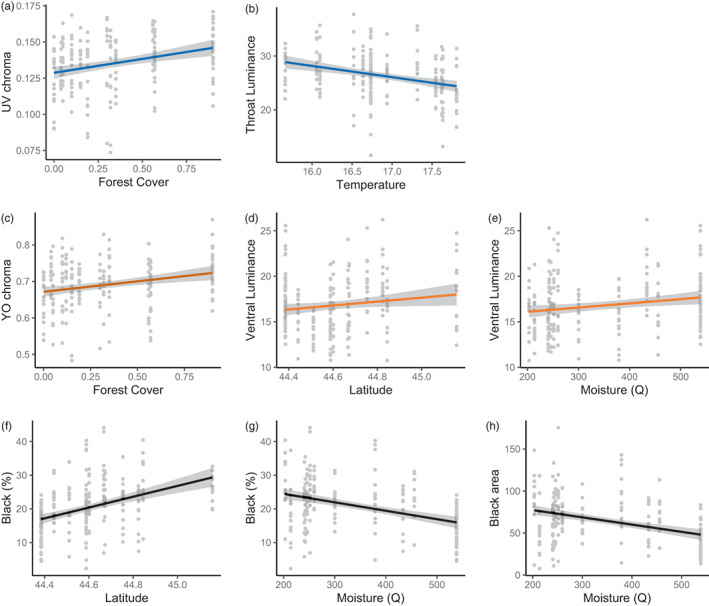
Linear regressions (based on raw data) between colour signal components and eco‐geographical variables, namely throat UV chroma with forest cover (a), throat luminance with mean annual temperature (b), ventral yellow‐orange chroma with forest cover (c), ventral luminance with latitude (d) and moisture (e), the percentage of black coloration with latitude (f) and moisture (g), and the absolute extent of black coloration with moisture (h). Blue, orange, and black regression lines refer the throat, belly and black coloration, respectively.

Regarding the effects of ecogeographical variables on lizard colouration (summarised in Table 1, Figure 4), in populations with higher forest cover, male common lizards have significantly higher throat UV chroma and higher ventral yellow–orange chroma. In addition, throat luminance decreases when mean annual temperatures increase, and ventral luminance increases at higher latitude and in more mesic habitats (i.e. higher values of *Q*). Finally, male common lizards with higher absolute amounts of black colouration are found in less mesic conditions. We also found this effect when we used relative black variable, which further correlates positively with latitude.

**TABLE 1:** Results from our conditional model averaging procedures examining the effect of ecogeographical variables on lizard colouration for each colour variable (six components). We provide the conditional averaged effect size estimates (standardised), standard errors, *p*‐values and importance value (i.e. sum of weights [sw]) associated with the effect of each ecogeographical predictor on each response colour variable. Statistically significant effects are in boldface and level of significance is indicated (*<0.05, ***<0.001).

Colour variable	Elevation	Latitude	Forest cover	Moisture	T_mean_
Throat UV chroma	−0.041 ± 0.145 *p* = 0.805 sw = 0.28	0.211 ± 0.136 *p* = 0.170 sw = 0.55	**0.347 ± 0.154** *p* = 0.045* **sw = 0.77**	−0.068 ± 0.157 *p* = 0.700 sw = 0.29	−0.022 ± 0.131 *p* = 0.885 sw = 0.26
Throat luminance	−0.124 ± 0.106 *p* = 0.312 sw = 0.62	0.141 ± 0.085 *p* = 0.146 sw = 0.43	0.108 ± 0.099 *p* = 0.332 sw = 0.43	−0.005 ± 0.096 *p* = 0.965 sw = 0.28	**−0.339 ± 0.091** *p* = 0.001*** **sw = 1.00**
Ventral Y‐O chroma	0.122 ± 0.125 *p* = 0.377 sw = 0.41	−0.085 ± 0.100 *p* = 0.449 sw = 0.35	**0.223 ± 0.094** *p* = 0.038* **sw = 0.77**	−0.086 ± 0.110 *p* = 0.490 sw = 0.36	−0.187 ± 0.087 *p* = 0.061 sw = 0.72
Ventral luminance	0.212 ± 0.112 *p* = 0.099 sw = 0.42	**0.294 ± 0.106** *p* = 0.014* **sw = 0.91**	−0.056 ± 0.133 *p* = 0.712 sw = 0.29	**0.275 ± 0.121** *p* = 0.045* **sw = 0.84**	−0.127 ± 0.124 *p* = 0.360 sw = 0.71
Black (absolute)	0.175 ± 0.144 *p* = 0.289 sw = 0.44	0.216 ± 0.136 *p* = 0.161 sw = 0.55	−0.132 ± 0.168 *p* = 0.488 sw = 0.33	**−0.340 ± 0.154** *p* = 0.049* **sw = 0.76**	−0.072 ± 0.147 *p* = 0.665 sw = 0.31
Black (%)	0.106 ± 0.132 *p* = 0.483 sw = 0.35	**0.280 ± 0.121** *p* = 0.040* **sw = 0.81**	−0.075 ± 0.155 *p* = 0.669 sw = 0.29	**−0.311 ± 0.139** *p* = 0.047* **sw = 0.78**	−0.104 ± 0.122 *p* = 0.453 sw = 0.36

## 4 DISCUSSION

Holistic studies integrating both environmental factors and individual traits as potential drivers of colour signal variation are scarce, and yet they have the potential to bring invaluable insights into the complexity of signalling systems. For example, Moreno‐Rueda et al. (2021) showed that proxies of environmental conditions and individual quality predicted variation of two distinct colour signals in the lacertid lizard *Psammodromus algirus*, in directions comparable to our results. Here, we found that the three different colour signals covary with different aspects of male quality, thus supporting the ‘multiple message’ hypothesis, and to a lesser degree the ‘backup’ hypothesis (Johnstone, 1996). The yellow–orange ventral colouration correlates negatively with tick load, thus indicating good health, and positively with bite force, a performance trait involved in lizards' male–male competitive interactions (e.g. Husak et al., 2006) and male sexual harassment towards females (Fitze et al., 2005). In addition, the absolute and relative black colouration area increases with body size and bite force, respectively, confirming previous findings obtained in a separate, oviparous lineage of this species (San‐Jose et al., 2017). Furthermore, the throat UV colouration of males correlated (albeit weakly based on values of the coefficient of determination) with maximal sprint speed, a trait important for fitness in lizards (Miles, 2004). This result is consistent with the role of UV signals previously identified in this species during male–male competition (Kawamoto et al., 2021; Martin et al., 2016) and female mate choice (Badiane et al., 2020). Our study thus indicates that receivers can extract information about multiple aspects of male condition including locomotor performance, bite force and parasite infestation from several components of the colouration of adult male common lizards.

Contrary to our initial predictions, T does not seem play a central role in colour signal variation as it had no direct effect on colouration, but influenced colour signal variation only indirectly through its effects on parasitism. We found a dual effect of T levels on parasite load, decreasing tick prevalence while increasing haemogregarines prevalence at the same time. Fuxjager et al. (2011) also found contrasting effects of plasma T levels on parasitism in the lizard *Sceloporus jarrovii*, but with a different pattern since it increased mite load, decreased gastrointestinal nematodes and had no effect on blood parasites. Some studies showed a positive association between T and parasite load (e.g. Olsson et al., 2000) while others found no correlations at all (e.g. Oppliger et al., 2004). Therefore, it seems that the relationship between T levels, parasitism and immunity is more complex than proposed by the ICHH (Folstad & Karter, 1992). It may be because the relationship between T and the immune system can be complex in itself, for example, depressing only adaptive immunity while reinforcing innate immunity (Ezenwa et al., 2012). Also, T can affect parasitism through its independent effect on behaviour, for example, increasing male sexual activity which might favour infestation by ectoparasites but not necessarily by endoparasites (e.g. Olsson et al., 2000). Finally, parasite load may be explained by other factors than T such as environmental conditions. For example, Wu et al. (2019) showed that, in common lizards, tick infestation decreased with elevation and vegetation cover, and increased with human disturbances and the presence of livestock.

In addition, tick prevalence had a negative effect on the yellow–orange signals while haemogregarine prevalence directly reduced male performance, which in turn reduced yellow–orange chroma. This phenotypic correlation is superficially consistent with the predictions of the Hamilton–Zuk hypothesis (Hamilton & Zuk, 1982), which assumes an association between bright colour signals and low parasite load. However, exploring the genetic mechanisms involved would be necessary to draw a firm conclusion because Hamilton–Zuk hypothesis posits a genetic correlation between sexual ornaments and resistance to parasites (Balenger & Zuk, 2014). In contrast, parasite load was uncorrelated with UV colouration, diverging from previous results showing either positive or negative correlations between parasite load and UV signals in other lacertid species (e.g. Megía‐Palma et al., 2016a, 2016b).

Our results further indicate that high endurance reduces yellow–orange luminance. Previous evidence in lacertid lizards, including in our species, suggests that a high concentration of carotenoid pigments in the skin reduces luminance (Megía‐Palma et al., 2021) and produces red‐shifted hues (Fitze et al., 2009). In light of this, our findings may reflect an effect of endurance on hue, which in this case ranges from yellow to orange. Because the reflectance peak of a yellow hue is located at lower wavelengths than for an orange hue, the total luminance of orange colour is generally lower than for yellow. This would mean that males with orange‐shifted hues have higher endurance capacities than males with yellow‐shifted hues (Sinervo et al., 2007), which would make sense because male common lizards gradually turn from yellow to orange as they age (Bonnaffé et al., 2018). Alternatively, a less parsimonious hypothesis may posit that a high endurance capacity constrains yellow–orange luminance. The carotenoid‐based colouration of common lizards appears to be correlated with tocopherols (i.e. vitamin E) availability and dietary lipids such that high dietary lipid uptake reduces vitamin E circulating levels, which in turn reduces carotenoid‐based colour expression (San‐Jose et al., 2012). Thus, we could speculate that a high endurance requires a high dietary lipid uptake, therefore constraining yellow–orange luminance (San‐Jose et al., 2012). Another explanation may rely on previous evidence showing that iridophores, and not carotenoids, can account for the variation of the yellow–orange ventral colouration of common lizards (San‐Jose et al., 2013), in which case the above hypotheses would not be plausible. In any case, studies investigating the physiological basis of the relationship between this colour trait and endurance are needed to test these hypotheses.

For a signal to be successful, not only should it make honest information available to the receivers but it must also transmit well through the environment towards the receiver (Bradbury & Vehrencamp, 2011). Variation in colour signals therefore reflects to some extent variation in the environmental light conditions, which depend on factors such as habitat structure and climatic conditions, so as to maximise signal detectability (Fleishman et al., 2020). Here, we first showed that the degree of forest cover negatively impacted the amount of average incoming light (i.e. irradiance), and especially in the UV range. Interestingly, both UV and yellow–orange signals of male lizards increased in chroma in populations with higher forest cover, that is in habitats characterised by degraded ambient light conditions. We suggest that these highly saturated colour signals are favoured by natural selection in these habitats to compensate for reduced light conditions and increase signal detectability. Along the same lines, Leal and Fleishman (2004) found that the lizard *Anolis cristatellus* had a brighter dewlap in mesic forests and a darker dewlap in xeric forests, which in both cases increased dewlap detectability. Comparable correlations between signal features and environmental parameters enhancing signal detectability have been shown in other taxa such as birds and fishes, and with other signalling modes such as acoustic and chemical signals (see Cummings & Endler, 2018 and references therein).

We also found that the three types of colour signals co‐varied with latitude and local climatic conditions. Surprisingly, these geographic gradients do not support, and even oppose the predictions from the thermal melanism hypothesis (Clusella Trullas et al., 2007) and from the revised version of the ‘Gloger's rule’ (Delhey, 2019). With the exception of the relative amount of black colouration, we indeed found that lizards from populations at higher latitudes, in more humid conditions and with lower temperatures had generally brighter throat and belly colouration with fewer black spots (Table 1, Figure 4). This unexpected finding suggests that mesic conditions promote generally brighter colour signals. Our results allow us to propose several hypotheses. First, more mesic habitats usually have higher cloud cover on average and denser vegetation, thus degrading ambient light conditions (e.g. Thorpe, 2002). Therefore, selection on signal efficacy may increase overall signal conspicuousness to optimise its detectability. Second, these mesic conditions may offer more favourable conditions, for example, by relaxing potential constraints such as predation and parasitism or by providing more resources, allowing common lizards to increase investment in sexual signals. For instance, the biology of this species is highly dependent on access to water, a resource that may be more abundant in more mesic conditions (see Rozen‐Rechels et al., 2021 and references therein). Altogether, these analyses suggest that habitat constraints on signal efficacy and climatic conditions explain colour signal variation across populations in this species, even though *c*. 80% of the variance is left unexplained in our model.

In summary, our study shows that multiple colour signals with different production mechanisms vary in how they relate to their information content and to the local light conditions. The three colour signals indeed exhibit condition‐dependent variation, involving performance, morphology and parasitism, suggesting that they convey honest information about different aspects of male condition. However, despite its effect on parasitism, T does not seem to play a central role as a mediator between colour signals and their information content, thus contradicting the immunocompetence handicap hypothesis (Folstad & Karter, 1992). At the same time, our results suggest that both UV and yellow–orange signals respond to variations in local light conditions so as to increase signal efficacy, and that all three colour signals respond to climatic conditions in a direction ruling out thermal melanism. Our integrative study demonstrates that individual quality traits and environmental conditions simultaneously explain the variations of multiple colour signals, and that the nature of these relationships is conditional on colour production mode.

## AUTHOR CONTRIBUTIONS

Arnaud Badiane and Jean‐François Le Galliard conceived the study; Arnaud Badiane, Andréaz Dupoué, Pauline Blaimont, Donald B. Miles, Anthony L. Gilbert and Jean Clobert performed fieldwork and collected the raw data; Arnaud Badiane, Mathieu Leroux‐Coyau, Anna Kawamoto and David Rozen‐Rechels processed the data; Arnaud Badiane performed the statistical analyses; Jean‐François Le Galliard and Sandrine Meylan supervised and commented each step of the process; Arnaud Badiane and Jean‐François Le Galliard wrote the manuscript and all authors provided invaluable feedback on the manuscript.

## CONFLICT OF INTEREST

The authors have no conflict of interest to declare.

## Supporting information


Appendix S1

## Data Availability

Data and code used in this study are freely available from the public repository Zenodo https://doi.org/10.5281/zenodo.6683661 (Badiane et al., 2022).
