# Supplementary material for: Environmental conditions and male quality traits simultaneously explain variation of multiple colour signals in male lizards
Source: J Anim Ecol. 2022 Aug 2;91(9):1906–17. doi: 10.1111/1365-2656.13773 (PMC9542398; doi:10.1111/1365-2656.13773)
Supplement: Supplementary file 1 — Appendix S1 [file JANE-91-1906-s001.docx]

**Environmental conditions and male quality traits simultaneously explain variation of multiple colour signals in male lizards**

Arnaud Badiane^1^, Andréaz Dupoué^1^, Pauline Blaimont^2^, Donald B. Miles^3^, Anthony L. Gilbert^3^, Mathieu Leroux-Coyau^1^, Anna Kawamoto^1^, David Rozen-Rechels^4^, Sandrine Meylan^1^, Jean Clobert^5^, Jean-François Le Galliard^1,6^

**Supplementary Information**

**Supp. Info. S1:** Localisation, elevation, mean annual temperatures (T), and number of males captured (*N*) from the 13 natural populations of the common lizard (*Z. vivipara*) in the Massif Central, France.

| **Population** | **Coordinates** | **Elevation (m)** | **T (°C)** | ***N*** |
| --- | --- | --- | --- | --- |
| BAR | 44.442234; 3.618474 | 1403 | 16.06 | 13 |
| BEL | 44.672442; 4.024956 | 1416 | 16.52 | 11 |
| BES | 44.587643; 3.508088 | 1227 | 17.81 | 16 |
| BOB | 44.823958; 4.228239 | 1433 | 15.66 | 16 |
| BOU | 44.753746; 3.515384 | 1391 | 16.64 | 15 |
| CARM | 45.158041; 2.838223 | 1211 | 17.32 | 13 |
| COM | 44.667354; 3.531928 | 1410 | 16.10 | 16 |
| LAJ | 44.844330; 3.431061 | 1325 | 16.94 | 12 |
| MON | 44.511183; 4.008156 | 1049 | 17.62 | 15 |
| PAR | 44.601054; 3.559713 | 1396 | 17.63 | 14 |
| PIM | 44.384970; 3.873304 | 1420 | 16.73 | 25 |
| ROB | 44.384091; 3.877833 | 1411 | 16.73 | 29 |
| TIO | 44.588575; 3.107019 | 1275 | 17.55 | 16 |

**Supp. Info. S2**

We obtained reflectance spectra from the throat and belly of each male (two replicates per body region) using a JAZ diode-array spectrophotometer with a R400-7-UV/VIS reading-illumination probe (Ocean Optics Inc.) and a notebook computer running OceanView (Ocean Optics Inc.). We took reflectance readings in a darkened room using a PX2 light source (Ocean Optics Inc.) for full spectrum illumination. We recorded reflectance spectra relative to a white diffuse standard (WS1; Ocean Optics Inc.) and a dark reading. We set integration time to 30, scans to average to 10, and boxcar width to 10. For data acquisition, we hand-held the probe over the centre of the targeted colour patch with a 90° angle between the probe and the skin surface (i.e. coincident normal recording geometry, Anderson and Prager 2006). An entomological pin attached to the tip of the probe allowed us to maintain a constant distance of 3 mm between the tip of the probe and the skin surface. We always aimed the probe at a skin area larger than 1.5 mm in diameter that did not contain any black spot to avoid spectral contamination (Badiane, Pérez i de Lanuza, García‐Custodio, Carazo, & Font, 2017). We later processed spectral data in R v.3.3.2 (R Development Core Team 2017) using the package *pavo* 2.0 (Maia, Gruson, Endler, & White, 2019). We cropped each spectrum between 300-700 nm, smoothed them using a loess smooth span of 0.2, and averaged the two replicates recorded for each body region.

**Supp. Info. S3**

Model list of the initial piecewise SEM. We indicate the modelling method, response variables, predictors and random intercept used in each model. Variables in bold were added to the models based on the tests of d-separation. We also provide marginal (R^2^m) and conditional (R^2^c) R^2^ for each model. This initial model had a Fisher’s C statistic of 28.39 with a p-value of 0.55 and on 30 degrees of freedom, and was thus accepted as it fitted the data. This model had an AIC of 140.39 and a BIC of 300.54. GLMM = generalised linear mixed models; LMM = linear mixed models; YO-chroma = yellow-orange chroma. We used LMMs with the R package *nlme* (Pinheiro, Bates, DebRoy, Sarkar, & R Core Team, 2019) to analyse normally distributed variables, namely body size, endurance, bite force, and the five colour variables (i.e. UV chroma, yellow-orange chroma, throat and belly luminance, and ventral blackness), and GLMMs with the R package *lme4* (Bates, Maechler, Bolker, & Walker, 2015) to analyse variables for which we assumed a Poisson distribution, namely the number of ticks and haemogregarines. We added population as random intercept in all models, and we log-transformed endurance and testosterone to comply with models’ assumptions.

| **Method** | **Response** | **Predictors** | **Random** | **R^2^m** | **R^2^c** |
| --- | --- | --- | --- | --- | --- |
| GLMM | Haemogregarines | ~ logTestosterone | Population | 0.03 | 0.87 |
| GLMM | Ticks | ~ logTestosterone | Population | 0.06 | 0.74 |
| LMM | Body size | ~ logTestosterone | Population | 0.01 | 0.31 |
| LMM | logEndurance | ~ Body size + logTestosterone | Population | 0.02 | 0.27 |
| LMM | Bite force | ~ Body size + logTestosterone + Haemogregarines | Population | 0.59 | 0.61 |
| LMM | Black | ~ logEndurance + BF + Ticks + Haemogregarines + Body size | Population | 0.24 | 0.31 |
| LMM | YO-Chroma | ~ logEndurance + BF + Ticks + Haemogregarines | Population | 0.16 | 0.17 |
| LMM | Belly luminance | ~ logEndurance + BF + Ticks + Haemogregarines | Population | 0.08 | 0.28 |
| LMM | UV chroma | ~ logEndurance + BF + Ticks + Haemogregarines | Population | 0.03 | 0.34 |
| LMM | Throat luminance | ~ logEndurance + BF + Ticks + Haemogregarines | Population | 0.07 | 0.19 |

**Supp. Info. S4**

Table summarising the statistics from the LMMs run seprately to test the relationship between the colour variables and sprint speed. These models had the colour variables as response, sprint speed as predictor, and population as random intercept. We provide the estimates (β), standard errors (SE), p-values, and marginal and conditional R^2^ for each model. The level of significance is indicated (* < 0.05). YO chroma = yellow-orange chroma

| **Response** | **β ± SE** | **p-value** | **R^2^m** | **R^2^c** |
| --- | --- | --- | --- | --- |
| UV chroma | 0.02 ± 0.01 | 0.040 * | 0.04 | 0.25 |
| YO chroma | 0.01 ± 0.03 | 0.67 | < 0.01 | 0.09 |
| Throat luminance | 0.15 ± 1.54 | 0.925 | 0.00 | 0.19 |
| Belly luminance | -0.63 ± 1.17 | 0.591 | < 0.01 | 0.10 |
| Absolute black | 0.46 ± 11.30 | 0.968 | 0.00 | 0.15 |
| % black | -0.86 ± 2.97 | 0.773 | < 0.01 | 0.16 |

**Supp. Info. S5**

False discovery rates (FDR) of 15%, 20%, and 25% were calculated following the Benjamini-Hochberg procedure for the structural equation modelling procedure. We provide three different values of false discovery rate (Q) to explore how they affect our results, and new p-values are calculated for each FDR. New significance thresholds are indicated by *p*-values highlighted in red for a FDR of 15%, in blue for a FDR of 20%, and in green for a FDR of 25%.

| **Response** | **Predictor** | ***P*-value** | **Rank** | **Q 15%** | **Q 20%** | **Q 25%** |
| --- | --- | --- | --- | --- | --- | --- |
| Haemegregarines | logTestosterone | 0.001 | 1 | 0.003 | 0.005 | 0.006 |
| Ticks | logTestosterone | 0.001 | 2 | 0.007 | 0.009 | 0.011 |
| Bite Force | Body Size | 0.001 | 3 | 0.010 | 0.014 | 0.017 |
| Absolute Black | Body Size | 0.001 | 4 | 0.014 | 0.018 | 0.023 |
| YO Chroma | Ticks | 0.001 | 5 | 0.017 | 0.023 | 0.028 |
| YO Luminance | Absolute Black | 0.003 | 6 | 0.020 | 0.027 | 0.034 |
| UV Luminance | UV Chroma | 0.004 | 7 | 0.024 | 0.032 | 0.040 |
| YO Chroma | Bite Force | **0**.**010** | 8 | 0.027 | 0.036 | **0.045** |
| UV Luminance | YO Luminance | 0.011 | 9 | 0.031 | 0.041 | 0.051 |
| YO Luminance | logEndurance | 0.018 | 10 | 0.034 | 0.045 | 0.057 |
| YO Luminance | UV Chroma | **0**.**020** | 11 | 0.038 | **0.050** | 0.063 |
| Bite Force | Haemogregarines | 0.026 | 12 | 0.041 | 0.055 | 0.068 |
| Absolute Black | UV Chroma | 0.026 | 13 | 0.044 | 0.059 | 0.074 |
| Relative Black | UV Chroma | **0**.**031** | 14 | **0**.**048** | 0.064 | 0.080 |
| UV Luminance | logEndurance | 0.057 | 15 | 0.051 | 0.068 | 0.085 |
| Haemegregarines | Body Size | 0.068 | 16 | 0.055 | 0.073 | 0.091 |
| UV Luminance | Ticks | 0.085 | 17 | 0.058 | 0.077 | 0.097 |
| YO Luminance | Ticks | 0.086 | 18 | 0.061 | 0.082 | 0.102 |
| Absolute Black | YO Chroma | 0.088 | 19 | 0.065 | 0.086 | 0.108 |
| YO Chroma | logEndurance | 0.089 | 20 | 0.068 | 0.091 | 0.114 |
| Bite Force | logEndurance | 0.095 | 21 | 0.072 | 0.095 | 0.119 |
| Relative Black | YO Chroma | 0.108 | 22 | 0.075 | 0.100 | 0.125 |
| YO Chroma | UV Chroma | 0.112 | 23 | 0.078 | 0.105 | 0.131 |
| logEndurance | Body Size | 0.117 | 24 | 0.082 | 0.109 | 0.136 |
| YO Luminance | Haemogregarines | 0.120 | 25 | 0.085 | 0.114 | 0.142 |
| Bite Force | logTestosterone | 0.122 | 26 | 0.089 | 0.118 | 0.148 |
| UV Luminance | YO Chroma | 0.137 | 27 | 0.092 | 0.123 | 0.153 |
| Absolute Black | logEndurance | 0.145 | 28 | 0.095 | 0.127 | 0.159 |
| UV Chroma | Haemogregarines | 0.190 | 29 | 0.099 | 0.132 | 0.165 |
| UV Luminance | Absolute Black | 0.190 | 30 | 0.102 | 0.136 | 0.170 |
| UV Chroma | Bite Force | 0.206 | 31 | 0.106 | 0.141 | 0.176 |
| Absolute Black | Ticks | 0.209 | 32 | 0.109 | 0.145 | 0.182 |
| UV Luminance | Haemogregarines | 0.214 | 33 | 0.113 | 0.150 | 0.188 |
| UV Luminance | Bite Force | 0.216 | 34 | 0.116 | 0.155 | 0.193 |
| Absolute Black | Bite Force | 0.235 | 35 | 0.119 | 0.159 | 0.199 |
| UV Chroma | Ticks | 0.299 | 36 | 0.123 | 0.164 | 0.205 |
| Body Size | logTestosterone | 0.311 | 37 | 0.126 | 0.168 | 0.210 |
| YO Luminance | YO Chroma | 0.394 | 38 | 0.130 | 0.173 | 0.216 |
| Haemegregarines | Ticks | 0.401 | 39 | 0.133 | 0.177 | 0.222 |
| logEndurance | logTestosterone | 0.709 | 40 | 0136 | 0.182 | 0.227 |
| YO Chroma | Haemogregarines | 0.732 | 41 | 0.140 | 0.186 | 0.233 |
| YO Luminance | Bite Force | 0.914 | 42 | 0.143 | 0.191 | 0.239 |
| UV Chroma | logEndurance | 0.943 | 43 | 0.147 | 0.195 | 0.244 |
| Absolute Black | Haemogregarines | 0.971 | 44 | 0.150 | 0.200 | 0.250 |

**Supp. Info. S6**

Best models (ΔAICc < 2) from the model selection procedure regarding the analysis of the relationship between ecogeographical variables and colouration. We provide the predictors, the AIC score, the ΔAICc, the weight, and the adjusted R^2^ of each model.

| **Response** | **Predictors** | **AICc** | **ΔAICc** | **Weight** | **R^2^** |
| --- | --- | --- | --- | --- | --- |
| Throat  UV chroma | Elevation + Latitude + Forest Cover | 532.1 | 0.00 | 0.286 | 0.139 |
|  | Latitude + Forest Cover | 533.6 | 1.50 | 0.135 | 0.122 |
|  | Elevation + Latitude + Forest Cover + Tmean | 533.7 | 1.60 | 0.129 | 0.142 |
|  | Elevation + Latitude + Forest Cover + Dryness | 533.9 | 1.86 | 0.113 | 0.141 |
| Throat luminance | Latitude + Tmean | 575.9 | 0.00 | 0.145 | 0.114 |
|  | Latitude + Forest Cover + Tmean | 576.3 | 0.39 | 0.120 | 0.122 |
|  | Elevation + Latitude + Forest Cover + Tmean | 576.4 | 0.51 | 0.113 | 0.131 |
|  | Tmean | 576.5 | 0.60 | 0.108 | 0.102 |
|  | Elevation + Tmean | 577.0 | 1.03 | 0.087 | 0.110 |
|  | Elevation + Latitude + Tmean | 577.4 | 1.44 | 0.071 | 0.117 |
|  | Latitude + Dryness + Tmean | 577.9 | 1.97 | 0.054 | 0.115 |
| Belly  Y-O chroma | Forest Cover + Tmean | 584.5 | 0.00 | 0.185 | 0.100 |
|  | Forest Cover + Dryness + Tmean | 585.5 | 0.96 | 0.114 | 0.105 |
|  | Latitude + Forest Cover + Tmean | 586.4 | 1.83 | 0.074 | 0.101 |
| Belly  Luminance | Elevation + Latitude + Dryness | 572.4 | 0.00 | 0.235 | 0.163 |
|  | Latitude + Dryness + Tmean | 573.9 | 1.46 | 0.113 | 0.157 |
|  | Elevation + Latitude + Forest Cover + Dryness | 574.1 | 1.65 | 0.103 | 0.165 |
|  | Elevation + Latitude + Dryness + Tmean | 574.3 | 1.86 | 0.093 | 0.164 |
| Black  (absolute) | Elevation + Latitude + Dryness | 549.7 | 0.00 | 0.097 | 0.255 |
|  | Latitude + Dryness | 549.9 | 0.16 | 0.090 | 0.246 |
|  | Elevation + Dryness | 550.1 | 0.33 | 0.083 | 0.246 |
|  | Dryness | 550.2 | 0.43 | 0.078 | 0.237 |
|  | Latitude | 550.6 | 0.90 | 0.062 | 0.235 |
|  | Dryness + Tmean | 550.8 | 1.08 | 0.056 | 0.243 |
|  | Elevation + Forest Cover + Dryness | 550.9 | 1.14 | 0.055 | 0.251 |
|  | Latitude + Dryness + Tmean | 551.1 | 1.31 | 0.050 | 0.250 |
|  | Elevation + Latitude + Forest Cover + Dryness | 551.5 | 1.73 | 0.041 | 0.257 |
| Black  (%) | Latitude + Dryness | 544.1 | 0.00 | 0.162 | 0.268 |
|  | Elevation + Latitude + Dryness | 544.7 | 0.58 | 0.121 | 0.274 |
|  | Latitude + Dryness + Tmean | 544.8 | 0.63 | 0.118 | 0.274 |
|  | Latitude | 545.7 | 1.56 | 0.074 | 0.254 |
| Black  (residuals) | Latitude + Dryness + Tmean | 554.8 | 0.00 | 0.198 | 0.235 |
|  | Latitude + Dryness | 555.0 | 0.21 | 0.179 | 0.226 |
|  | Elevation + Latitude + Dryness | 556.1 | 1.29 | 0.104 | 0.230 |
|  | Latitude + Forest Cover + Dryness + Tmean | 556.4 | 1.61 | 0.089 | 0.238 |
|  | Latitude + Forest Cover + Dryness | 556.6 | 1.79 | 0.081 | 0.228 |

**Supp. Info. S7**

False discovery rates (FDR) of 15%, 20%, and 25% were calculated following the Benjamini-Hochberg procedure for the model averaging procedure. We provide three different values of false discovery rate (Q) to explore how they affect our results, and new p-values are calculated for each FDR. Significance thresholds are indicated as bold *p*-values highlighted in red for a FDR of 15%, in blue for a FDR of 20%, and in green for a FDR of 25%.

| **Response** | **Predictor** | **P** | **Rank** | **Q 10%** | **Q 20%** | **Q 25%** |
| --- | --- | --- | --- | --- | --- | --- |
| UV Chroma | Forest Cover | 0.000 | 1 | 0.005 | 0.007 | 0.008 |
| UV Luminance | Temperature | 0.001 | 2 | 0.010 | 0.013 | 0.017 |
| YO Luminance | Latitude | 0.014 | 3 | 0.015 | 0.020 | 0.025 |
| YO Chroma | Forest Cover | 0.038 | 4 | 0.020 | 0.027 | 0.033 |
| UV Chroma | Latitude | 0.039 | 5 | 0.025 | 0.033 | 0.042 |
| Relative Black | Latitude | **0.040** | 6 | 0.030 | 0.040 | **0.050** |
| YO Luminance | Dryness | 0.045 | 7 | 0.035 | **0.047** | 0.058 |
| Relative Black | Dryness | 0.047 | 8 | 0.040 | 0.053 | 0.067 |
| Absolute Black | Dryness | **0.050** | 9 | **0.045** | 0.060 | 0.075 |
| YO Chroma | Temperature | 0.061 | 10 | 0.050 | 0.067 | 0.083 |
| YO Luminance | Elevation | 0.099 | 11 | 0.055 | 0.073 | 0.092 |
| UV Chroma | Elevation | 0.140 | 12 | 0.060 | 0.080 | 0.100 |
| UV Luminance | Latitude | 0.146 | 13 | 0.065 | 0.087 | 0.108 |
| Absolute Black | Latitude | 0.161 | 14 | 0.070 | 0.093 | 0.117 |
| Absolute Black | Elevation | 0.289 | 15 | 0.075 | 0.100 | 0.125 |
| UV Luminance | Elevation | 0.312 | 16 | 0.080 | 0.107 | 0.133 |
| UV Luminance | Forest Cover | 0.332 | 17 | 0.085 | 0.113 | 0.142 |
| YO Luminance | Temperature | 0.360 | 18 | 0.090 | 0.120 | 0.150 |
| YO Chroma | Elevation | 0.377 | 19 | 0.095 | 0.127 | 0.158 |
| YO Chroma | Latitude | 0.449 | 20 | 0.100 | 0.133 | 0.167 |
| Relative Black | Temperature | 0.453 | 21 | 0.105 | 0.140 | 0.175 |
| Relative Black | Elevation | 0.483 | 22 | 0.110 | 0.147 | 0.183 |
| Absolute Black | Forest Cover | 0.488 | 23 | 0.115 | 0.153 | 0.192 |
| YO Chroma | Dryness | 0.490 | 24 | 0.120 | 0.160 | 0.200 |
| Absolute Black | Temperature | 0.665 | 25 | 0.125 | 0.167 | 0.208 |
| Relative Black | Forest Cover | 0.669 | 26 | 0.130 | 0.173 | 0.217 |
| YO Luminance | Forest Cover | 0.712 | 27 | 0.135 | 0.180 | 0.225 |
| UV Chroma | Temperature | 0.803 | 28 | 0.140 | 0.187 | 0.233 |
| UV Chroma | Dryness | 0.891 | 29 | 0.145 | 0.193 | 0.242 |
| UV Luminance | Dryness | 0.965 | 30 | 0.150 | 0.200 | 0.250 |

**Supp. Info. S8**

Best selected path diagram representing the relationships between colour signals (bottom line, 5 colour components including the proportion of black colouration instead of the absolute amount of black), body size (measured by snout-vent length), physiological performance (bite force and endurance), parasitism (number of ticks and blood haemogregarines) and plasma testosterone levels. Each single-headed arrow represents a statistically significant direct causal path and arrow thickness is proportional to their effect size. Significant correlations are indicated by doubleheader arrows.


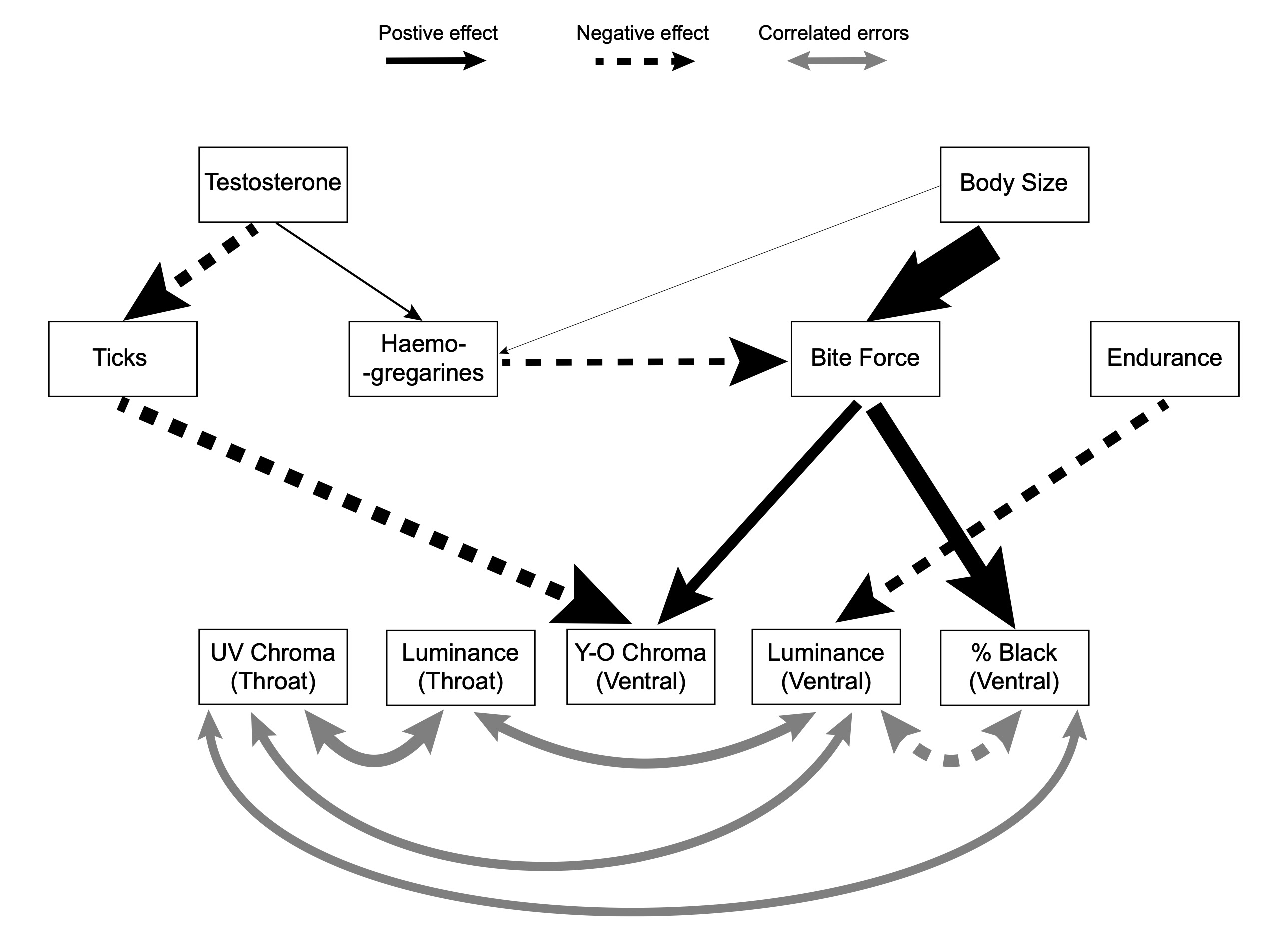


**References**

Anderson, S., & Prager, M. (2006). Quantifying colors. In G. E. Hill & K. J. Mcgraw (Eds.), *Bird coloration: Volume 1, mechanisms and measurements* (pp. 41–89).

Badiane, A., Pérez i de Lanuza, G., García‐Custodio, M. del C., Carazo, P., & Font, E. (2017). Colour patch size and measurement error using reflectance spectrophotometry. *Methods in Ecology and Evolution*, *8*(11), 1585–1593. doi: 10.1111/2041-210X.12801

Bates, D., Maechler, M., Bolker, B., & Walker, S. (2015). Fitting linear mixed-effects models using lme4. *Journal of Statistical Software*, *67*(1), 1–48.

Maia, R., Gruson, H., Endler, J. A., & White, T. E. (2019). pavo 2: New tools for the spectral and spatial analysis of colour in r. *Methods in Ecology and Evolution*, *10*(7), 1097–1107. doi: 10.1111/2041-210X.13174

Pinheiro, J., Bates, D., DebRoy, S., Sarkar, D., & R Core Team. (2019). *nlme: Linear and nonlinear mixed effects models*. R package version 3.1-141.

RDevelopmentCoreTeam. (2017). *R: A language and environment for statistical computing*. Vienna, Austria: R Foundation for Statistical Computing.
